# Efficacy of Inotuzumab Ozogamicin plus Ponatinib Followed by Allogeneic Stem Cell Transplantation in a Patient with Relapsed Philadelphia Chromosome-Positive Acute Lymphoblastic Leukemia

**DOI:** 10.1155/2021/1717506

**Published:** 2021-05-26

**Authors:** Ilina Micheva, Vladimir Gerov, Stela Dimitrova, Merlin Efraim

**Affiliations:** UMHAT “Sveta Marina” Medical University, Varna, Bulgaria

## Abstract

Philadelphia chromosome-positive acute lymphoblastic leukemia (Ph + ALL) is an aggressive disease with poor outcomes. Despite the incorporation of tyrosine kinase inhibitors (TKIs) in the therapeutic strategies, patients who relapse after chemotherapy plus TKI have poor overall survival (OS) and less chance to proceed to hematopoietic stem cell transplantation (HSCT) which remains the only curative approach. Therefore, new drugs, such as antibody-targeted therapies alone or in combination with TKIs, offer new therapeutic options for those patients. However, the combination of inotuzumab plus ponatinib has limited application. We present a case of a patient affected by Ph + ALL with T315I mutation successfully treated after early relapse with inotuzumab plus ponatinib, followed by allogeneic HSCT and ponatinib maintenance.

## 1. Introduction

Philadelphia chromosome (Ph) is the most common cytogenetic abnormality in adult patients with acute lymphoblastic leukemia (ALL), found in about 20–30% of ALL cases [1]. The incidence of Ph + ALL increases with age, reaching 43.8% in patients older than 50 years of age [2]. Patients with Ph + ALL have poor prognosis with higher rates of relapse and worse overall survival.

In the era of tyrosine kinase inhibitors (TKIs), the outcome of those patients has dramatically improved, and currently, the standard of care in the frontline setting is TKI in combination with chemotherapy [3,4]. However, there is still high risk of relapse, especially among patients with resistant BCR-ABL1 mutations [5]. Allogeneic hematopoietic stem cell transplantation (HSCT) is considered in the first remission, especially for younger patients treated with imatinib combination therapy [6].

Novel drugs, such as potent later-generation TKIs, antibody-drug conjugates, bispecific monoclonal antibodies, and chimeric antigen receptor T-cell (CAR T) therapies, are being developed and investigated in patients with Ph + ALL [7].

Inotuzumab and Blinatumumab have shown promising results in relapse/refractory (R/R) Ph + ALL [8,9]. Ponatinib, a third-generation TKI, has proven to be the most potent TKI for patients with Ph + ALL and the only one capable of overcoming the T315I mutation [10].

Recently, the combination of monoclonal antibodies (MoAbs) and TKI has been evaluated in Ph + ALL in frontline or relapse/refractory settings [11,12]. However, the combination of inotuzumab plus ponatinib has limited application. There is only one report where these novel drugs have been used sequentially in a patient with relapsed Ph + ALL. Pirosa MC et al. describe a case of a young patient with Ph′+ALL, who relapsed after second HSCT, who reached long-term disease control with molecular remission by treatment with inotuzumab ozogamicin, donor lymphocyte infusion, and ponatinib [13].

## 2. Materials and Methods

We present a case of a patient affected by Ph + ALL with T315I mutation successfully treated after early relapse with inotuzumab plus ponatinib, followed by allogeneic HSCT and ponatinib maintenance.

## 3. Results

Our case represents a 54-year-old female who was was referred to our hospital in June 2019 with fatigue, petechiae on the lower limbs, splenomegaly, and abnormal complete blood count (CBC) with hemoglobin 8.7 g/dL, platelets 89 × 10^9^/L, and white blood cell 72.01 × 10^9^/L. The morphological exam of the peripheral blood and bone marrow showed about 90% lymphoid blast cells.

The flow cytometric analysis confirmed the diagnosis of precursor B-cell ALL with following phenotype: CD19++/+++, CD22++, CD10++/+++, HLA-DR++/+++, CD34++, CD38+/++, CD13+, CD33 low+, CD123+/++, CD71 low+, CD58++, CD20+, CD45 low+, cyCD79a+, and nTdT+. Conventional cytogenetic study of the bone marrow, using GTG banding, revealed the presence of the Ph chromosome in 80% of the metaphases (karyotype 46,XX,t(9;22) (q34;q11)) [14]. Additionally, a single metaphase with trisomy 8 and 9 was described (49,XX,+8,+9, t(9;22) (q34;q1), but considered insignificant. Molecular analysis by real-time PCR showed 0.0909% BCR-ABL Mbcr (b3a2, b2a2) transcripts.

Treatment with H-CVAD plus imatinib mesylate was initiated according to the protocol. After 2 courses of therapy, complete remission (CR) was achieved. Treatment continued with two more cycles, and the HSCT from HLA-identical sibling was planned for March 2020. A week before the planned admission to the transplant unit, the patient appeared for consultation asymptomatic with CBC, revealing extreme leukocytosis- 143.00 10^9^/L, normal hemoglobin- 135 g/L, and platelets- 125 10^9^/L. The morphology and immunophenotype were consistent with the initial diagnosis. The BCR-ABL/ABL transcript level was 40%. T315I mutation was detected.

In March 2020, inotuzumab therapy was initiated and administered according to the protocol: 0.8 mg/m^2^ i.v. on day +1 and 0.5 mg/m^2^ i.v. on days +8 and +15 as described by Kantarjian et al. [8]. Corticosteroids were given before the first infusion of inotuzumab in order to decrease the white blood cell count and decrease the risk of tumor lysis syndrome. Ponatinib in a dose of 30 mg daily was added with the first administration of inotuzumab. No serious toxicity grade 3 and 4 were registered. After the first cycle, the patient achieved complete CR with a deep molecular response (DMR) (BCR- ABL/ABL IS = 0.0023%, 4.5 log) and proceeded to HSCT.

In April 2020, HSCT was performed by a matched related donor using a myeloablative conditioning regimen (thiotepa 5 mg/kg for 2 days, fludarabine 30 mg/m^2^ for 5 days, and busilvex 3.2 mg/kg for 3 days) and mobilized peripheral blood stem cells (CD34+ 2.14 × 10^6/kg). Revaluation on day +30 revealed DMR (BCR- ABL/ABL IS = 0.0047%, 4.3 log). Ponatinib maintenance was initiated at a dose of 30 mg/day. The patient was followed up monthly and maintained DMR. On month 6, an asymptomatic increase of the liver enzymes was registered with ALT- 524.7 U/l (range 10–49 U/l), ASAT- 257.6 U/l (range 0–34 U/l), ALP- 358 U/l (range 45–129 U/l), GGT 208 U/l (range 0–38 U/l), and lactate dehydrogenase- 437 U/l (range 208–378 U/l). Infectious or autoimmune hepatitis was excluded, and there were no signs of graft versus host disease. Toxicity was suspected, and ponatinib was stopped. A month later, after treatment with ursodeoxycholic acid and silymarin, the liver indexes were normalized and Ponatinib was restarted at a dose of 15 mg/day.

At the time of reporting, the patient was asymptomatic, in sustained molecular response (BCR- ABL/ABL IS = 0.000097%, 6 log). Maintenance with ponatinib continues in a dose of 15 mg/day with excellent tolerability.

## 4. Discussion

Our case represents an early recurrence of ALL, with unfavourable T315 mutation, in which the possibility of using MoAb as a rescue therapy and bridge to HSCT was discussed. Both inotuzumab and blinatumomab have shown superiority over chemotherapy in terms of efficacy. The experience with inotuzumab in Ph + ALL patients comes from the clinical trials of inotuzumab. Among Ph + patients in the INO-VATE study, the rates of CR/CR incomplete, minimal residual disease (MRD) and subsequent HSCT were higher in the inotuzumab arm compared to standard chemotherapy [8].

Blinatumomab was tested in adults with R/R Ph + ALL in a phase 2 study ALCANTARA and showed a CR of 36%, including four of 10 patients with the T315I mutation, complete MRD response in 88% of CR responders, and a median OS of 7.1 months [9].

At the time of recurrence, blinatumomab was not approved for treatment of patients with Ph + ALL. Inotuzumab was applied based on the CD22 expression, the efficacy even in high tumour burden with >50% lymphoblasts in the bone marrow and taking into account the increased risk of veno-occlusive disease (VOD), reported as the major nonhematologic adverse event associated with inotuzumab [8].

It has been demonstrated that the presence of T315I mutation in Ph + ALL is associated with a highly aggressive disease phenotype and resistance to TKI therapy [5]. Analysis of 17 TKI-resistant Ph + leukemia patients who were found to have an ABL kinase domain mutation showed that combined use of different TKIs and complex chromosomal karyotypes may promote the development of the T315I mutation. Ratio of blast cells >50% and number of white blood cells >20 × 10^9^/L were related to poor survival prognosis [15].

Ponatinib is a third-generation TKI that is currently approved as per label when no other TKIs are indicated for the treatment of patients with CML and Ph + ALL after failing treatment with second-generation TKIs or if the presence of T315I mutation is discovered [14]. In the phase II PACE trial, among patients with Ph + ALL, 41% achieved a major hematologic response (MaHR) by 6 months with a median time to MaHR among responders 0.7 months (range, 0.4–5.5 months). The median duration of MaHR in responders with Ph^+^ ALL was 3.2 months (range, 1.8–12.8 months). Major cytogenetic response (CyR) and complete CyR were achieved by 47% and 38% of patients, respectively. Median progression-free survival (PFS) was 3.0 months, and OS at 3 years was 12%. A multivariate analysis showed that the presence of T315I itself is not a predictor of response [16].

Recently, several studies have evaluated the efficacy of MoAb in combination with TKI.

A retrospective study showed high efficacy of the combination blinatumomab and TKI (ponatinib, dasatinib, or bosutinib) in R/R Ph + ALL and chronic myeloid leukemia (CML) in lymphoid blast phase with molecular responses of 75% and one-year OS rates of 73%. Two of the patients in the analysed group were with T315I mutation [17].

The combination blinatumomab-dasatinib was evaluated in front-line settings in a phase 2 single-group trial in adults with newly diagnosed Ph + ALL. CR was observed in 98%, and after two cycles of blinatumomab, 60% of the patients had molecular response. At a median follow-up of 18 months, overall survival was 95% and disease-free survival was 88% [11].

The combined use of inotuzumab and ponatinib, both drugs highly effective in Ph^+^ ALL with T315I mutation, has been avoided due to high risk of hepatotoxicity. Efficacy of inotuzumab with less hepatotoxic TKI, particularly bosutinib, has been recently investigated. CR was detected in 50% of patients, with major molecular response (MMR) in 85%, and the OS was 0.7 months. Notably, patients with T315I mutation were excluded from this study [12].

Despite the limited experience and expected high liver toxicity, we used the combination inotuzumab plus ponatinib achieving CR and MRD negativity after one course of treatment, which allowed us to proceed to HSCT.

Maintenance with TKIs should be considered after transplant to reduce relapse rates in Ph + ALL. However, the choice of TKI and the duration of maintenance therapy remain uncertain. A systematic review, comparing survival outcomes of second-generation TKIs nilotinib and dasatinib with first-generation TKI imatinib after HSCT in Ph + ALL, showed that the use of all TKIs after allo-HSCT for patients in CR1 improved OS when given as a prophylactic or preemptive regimen. Limited data suggest that second-generation TKIs (i.e., dasatinib) have a better OS, especially in patients with MRD-positive status. Imatinib did not improve OS in patients who were >CR1 at the time of allo-HSCT [18].

In a recently published study, analyzing the impact of TKI maintenance after HSCT in Ph + ALL, 59% of patients received posttransplant TKI as prophylaxis or at the first MRD positivity (11 from 95 patients received ponatinib). Median time to TKI initiation after allo-HSCT was 2.4 months (range 19 days–35 months). The 2-year PFS for patients who initiated prophylaxis within 3 months after transplantation was 94.5% (95% CI: 74.3–99.0, med. PFS = 144 mo.) compared to 75% in patients who received TKI after 3 months of transplantation (95% CI: 46.8–82.0, med. PFS = 96 mo.) (*P* value = .041). In the TKI prophylactic group, the relapse rate was similar between patients receiving imatinib or newer-generation TKIs. In the MRD-triggered group, 75% of patients who received imatinib relapsed compared to 45% of patients who received newer-generation TKI. Newer-generation TKIs may overcome some of the unfavorable mutations and may lead to lower relapse rates in patients with more advanced disease [19].

During the course of ponatinib maintenance, we registered hepatotoxicity, requiring stopping of ponatinib and dose reduction.

Ponatinib was approved at the dose of 45 mg/day [16]. However, because of the close relationship between the dose of ponatinib and the risk of cardiovascular events, dose reduction was suggested. It has been demonstrated that each 15 mg reduction causes an approximate 33% reduction of the risk of arterial thrombosis with sustained antileukemic activity [20].

In the era of monoclonal antibodies and third-generation TKI, inotuzumab plus ponatinib appeared a potent combination inducing complete molecular response in an extremely high-risk Ph + ALL with T315I mutation. TKI maintenance after transplantation may play an important role decreasing the rate of hematological relapse and improving disease-free survival in such challenging and difficult-to-treat cases.

## Data Availability

No data were used to support this study.
